# Diffuse nuclear Overhauser effect MRI contrast changes detected in multiple sclerosis subjects at 7T

**DOI:** 10.1093/braincomms/fcaf043

**Published:** 2025-02-20

**Authors:** Paul S Jacobs, Anshuman Swain, Neil Wilson, Fang Liu, Blake Benyard, Bailey Spangler, Madeleine Seitz, Allen Fu, Ravi Prakash Reddy Nanga, Mark A Elliott, Amit Bar-Or, John Detre, Jennifer Orthmann Murphy, Matthew K Schindler, Ravinder Reddy

**Affiliations:** Center for Advanced Metabolic Imaging in Precision Medicine, Department of Radiology, University of Pennsylvania, Philadelphia, PA 19104, USA; Center for Advanced Metabolic Imaging in Precision Medicine, Department of Radiology, University of Pennsylvania, Philadelphia, PA 19104, USA; Center for Advanced Metabolic Imaging in Precision Medicine, Department of Radiology, University of Pennsylvania, Philadelphia, PA 19104, USA; Penn Statistics in Imaging and Visualization Center, Department of Biostatistics, Epidemiology, and Informatics, University of Pennsylvania, Philadelphia, PA 19104, USA; Center for Advanced Metabolic Imaging in Precision Medicine, Department of Radiology, University of Pennsylvania, Philadelphia, PA 19104, USA; Penn Statistics in Imaging and Visualization Center, Department of Biostatistics, Epidemiology, and Informatics, University of Pennsylvania, Philadelphia, PA 19104, USA; Penn Statistics in Imaging and Visualization Center, Department of Biostatistics, Epidemiology, and Informatics, University of Pennsylvania, Philadelphia, PA 19104, USA; Penn Statistics in Imaging and Visualization Center, Department of Biostatistics, Epidemiology, and Informatics, University of Pennsylvania, Philadelphia, PA 19104, USA; Center for Advanced Metabolic Imaging in Precision Medicine, Department of Radiology, University of Pennsylvania, Philadelphia, PA 19104, USA; Center for Advanced Metabolic Imaging in Precision Medicine, Department of Radiology, University of Pennsylvania, Philadelphia, PA 19104, USA; Department of Neurology, University of Pennsylvania, Philadelphia, PA 19104, USA; Center for Advanced Metabolic Imaging in Precision Medicine, Department of Radiology, University of Pennsylvania, Philadelphia, PA 19104, USA; Department of Neurology, University of Pennsylvania, Philadelphia, PA 19104, USA; Department of Neurology, University of Pennsylvania, Philadelphia, PA 19104, USA; Department of Neurology, University of Pennsylvania, Philadelphia, PA 19104, USA; Center for Advanced Metabolic Imaging in Precision Medicine, Department of Radiology, University of Pennsylvania, Philadelphia, PA 19104, USA

**Keywords:** magnetic resonance, multiple sclerosis, lipid metabolism, CEST, NOE

## Abstract

Multiple sclerosis is an inflammatory demyelinating condition of the central nervous system affecting approximately 1 million people in the USA. Although standard structural MRI techniques are now the main imaging modality for multiple sclerosis diagnosis and management, they are yet to provide information regarding the metabolic profile of the disease. Ultra-high field 7T MRI systems have provided gains in signal-to-noise ratio (SNR) and spatial resolution for structural MRI as well as larger chemical shifts leading to improvements in specialized imaging sequences, such as nuclear Overhauser effect (NOE) imaging, that can evaluate macromolecular metabolite composition. In this work, NOE images were acquired on a cohort of multiple sclerosis and healthy control subjects to spatially map differences in their lipid metabolites as a result of NOE effects. NOE image data were acquired on a total of 25 subjects {15 multiple sclerosis subjects [10 females, 5 males (21–70 years)] and 10 healthy controls [5 females, 5 males (23–71 years)]} on a 7T MRI system with a frequency offset range of −5 to 5 ppm. A five-pool Lorentzian line fitting model was utilized to fit and quantitatively compare direct saturation (DS), magnetization transfer (MT), amide proton transfer (APT), amine, and relayed NOE (rNOE) and used as a comparison to conventional T_1_ maps. Grey and white matter tissues were segmented using the T_1_ maps, while the lesion tissue was segmented manually. Correlations between disease duration and lesion load were performed to investigate any existing relationship to image contrast. The primary findings of this work include statistically significant decreases in the rNOE pool for the normal-appearing white matter (NAWM) (11.4% decrease) and normal-appearing grey matter (NAGM) (10.6% decrease) in multiple sclerosis subjects compared to healthy controls. Additionally, a significant decrease in the amine pool was also observed for NAWM (15.3% decrease) in multiple sclerosis subjects compared to healthy controls. Changes in multiple sclerosis lesion contrast were also observed for several pools (DS, amine, and rNOE). Decreases in both the rNOE and amine pools suggest that in multiple sclerosis, there are diffuse decreases in mobile lipids, such as those found in neuronal cell bodies, as well as a decrease in proteins with amine groups. Furthermore, these measurable contrast changes were not detected in the corresponding T_1_ maps. NOE imaging can provide complementary metabolic information to conventional MRI methods. Future studies will focus on utilizing this technique for longitudinal tracking of disease progression and investigating similar demyelinating diseases.

## Teaser

Metabolic MRI was performed at an ultra-high field to evaluate nuclear Overhauser effect lipid metabolite content in a cohort of multiple sclerosis subjects. Jacobs *et al*. report metabolite decreases in pathological white and grey matter. These diffuse decreases in mobile lipids and proteins suggest a potential metric for global tissue injury.

## Graphical Abstract

**graphical abstract:**
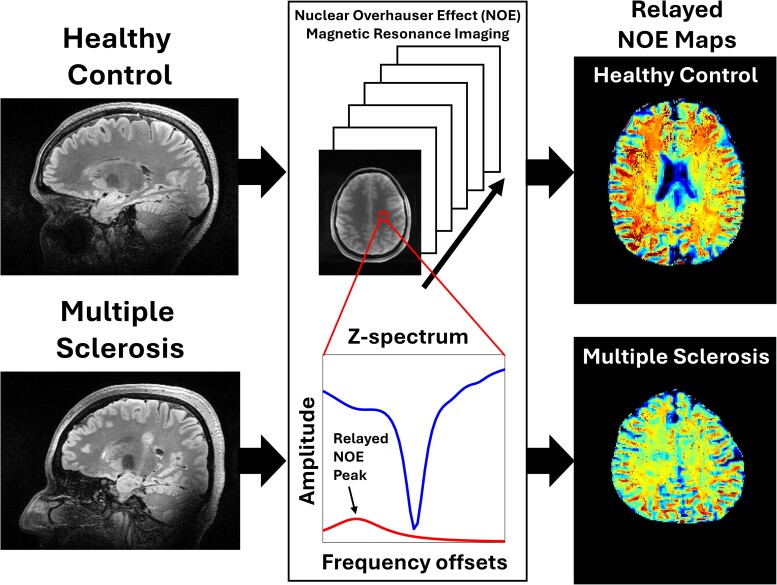

## Introduction

Multiple sclerosis is an immune mediate condition of the CNS that affects approximately 1 million people in the USA and is typically diagnosed in individuals between 20 and 30 years of age.^1,2^ In the past, multiple sclerosis has typically been categorized via distinct clinical course descriptions including relapsing–remitting, secondary progressive, and primary progressive. However, accumulating evidence has suggested that multiple sclerosis disease progression is better described as a continuum consisting of multiple mechanisms of injury (e.g. non-resolving inflammation, neurodegeneration, oxidative stress, and mitochondrial dysfunction) in combination with compensatory mechanism failures (e.g. remyelination and neuroplasticity).^3,4^ While the exact cause is unknown, multiple sclerosis pathogenesis is thought to stem from antigen-presenting cells (such as B cells, dendritic cells, microglia, and macrophages) triggering an adaptive immune response by activating CD4 + and CD8+ T cells. Multiple sclerosis disease progression can typically be thought of in two over-lapping processes: an acute inflammatory response and a chronic progressive inflammatory neurodegeneration.^5^ This complex biology can result in focal inflammatory demyelination and chronic, widespread neuroinflammation as well as neuronal degeneration. These processes can also lead to more advanced progressive forms of the disease typically accompanied by severe cognitive and motor impairment.^6^ Previous studies have suggested that these processes can result in two forms of inflammation, a focal lesional type and one with more diffuse involvement.^7^ Lipids have also been thought to play a significant role in these processes, not only due to the importance of lipids in myelin sheath maintenance, but also for their role in cell signalling, communication, and transportation within the CNS microenvironment.^8^ Improper regulation of important lipid metabolites, such as sphingolipids and ceramides, have been linked to oligodendrocyte damage as well as acute demyelination.^9^ Furthermore, the modulation of plasma membrane lipids such as cholesterol and glycosphingolipids can influence immune cell differentiation as well as function resulting in potential pathogenic consequences.^10^ However, while these lipid metabolism mechanisms have been well characterized in the lesion environment, their role within the diffuse normal-appearing tissue regions and how it influences multiple sclerosis disease processes has not been evaluated.

MRI is an essential tool for diagnosis and treatment monitoring in multiple sclerosis multiple sclerosis .^11^ Lesion identification primarily within the white matter (WM) have typically been performed using sequence such as FLAIR and MP2RAGE (with and without contrast enhancement) as hyperintense and hypointense contrast, respectively, on conventional 1.5T and 3T MR systems. These commonly used anatomical sequences primarily provide size- and location-based information, albeit with low specificity in terms of the metabolic underpinnings of the underlying pathology, particularly when involving lipid metabolism. The commonly used lower-field strength MR systems also have a practical upper limit in terms of resolution and signal-to-noise ratio.^12^ Additionally, more advanced sequences, such as myelin water imaging (MWI),^13^ have been gaining traction as a potential imaging biomarker for myelin; however, the signal from this method does not account for the large concentrations of lipids comprising the myelin sheath. Therefore, the MWI may not be the most robust method for detecting changes in myelin-associated lipids.

An ultra-high field MRI (≥7T) has allowed for significant increases in both resolution and signal-to-noise ratio, as well as providing a platform for using specialized chemical exchange saturation transfer (CEST)-based sequences due to an increase in chemical dispersion at higher-field strengths. The nuclear Overhauser effect (NOE) is one such techniques that has been used to evaluate macromolecules, such as lipids and proteins in the brain.^14^ This technique generate endogenous contrast by using magnetization transfer via dipolar-cross relaxation between saturated (lipids and proteins) and unsaturated (bulk water) spins systems.^15^ By repeating this measurement across a range of frequency values, a high-spectral resolution pixel-based Z-spectrum can be generated. This overall Z-spectral signal includes contribution from the exchange with various functional groups such as aliphatic, amide, and amine protons. Additionally, the saturation of protons in free (direct saturation) and restricted (magnetization transfer) water molecules simultaneously have large contributions to the overall signal. To separate out these various metabolite signals, previous studies have had success employing Lorentzian line fitting to generate a multi-pool model of the contributing signals.^16-18^

Prior relevant work by Huang *et al.*^19^ leveraged relayed NOE-weighted (rNOEw) imaging at 3T to study lipid/protein metabolism between a population of patients with multiple sclerosis, neuromyelitis optica spectrum disorder, and healthy control subjects. In this work, the authors observed significant decreases in both lesional and diffuse tissue contrast between healthy and multiple sclerosis subjects. However, this was done at 3T field strength and only the rNOEw signal was used as a primary imaging contrast, which limited conclusions about the underlying pathology. In a more recent study, O’Grady *et al.*^20^ imaged a population of multiple sclerosis subjects at 7T using glutamate-weighted CEST (GluCEST) MRI. In this work, changes were observed as an increase in contrast in the WM lesion, while normal-appearing tissue did not show substantial changes. However, since this study primarily focused on glutamate-weighted CEST as the main imaging metric, conclusions about the disease underpinnings were also limited.

Other techniques, such as MWI have been used as a proxy for direct myelin imaging due to the proton signal from the bound pool of water having T_2_ times much longer (∼10 ms) than the myelin lipid protons (∼50µs).^13,21^ An example of this technique’s utility was demonstrated by Laule *et al.,*^21^ in which the authors measured the short T_2_ component of bound myelin water to estimate the myelin water fraction within the diffuse WM. They found higher water content in the normal-appearing white matter (NAWM) and in multiple sclerosis lesions compared to healthy controls. They also found lower myelin water fraction in NAWM compared to healthy controls indicating a possible combination of diffuse oedema, chronic inflammation, and demyelination. However, lipid changes can occur prior to alteration in the myelin bi-layer water, so a technique sensitive to the lipid pool may provide earlier diagnostic information. Some evidence also suggests that the movement of water from myelin during the measurement can occur relatively quickly to cause an artificial reduction in the measuredmyelin water fraction.^21^ Inhomogeneous magnetization transfer is a technique that also has been used to provide myelin sensitive contrast by exploiting the observed asymmetry of the magnetization transfer (MT) spectral bound pool (primarily drive by dipolar effects) which gives highly restricted protons in lipid chains (i.e. myelin) more contrast sensitivity. Additionally, quantitative magnetization transfer uses a multi-compartment model to systematically vary the saturation offset and power to derive parameters such as pool size ratio, macromolecular content, and macromolecular proton fraction which can provide information on myelin content.^22^ However, because these techniques are all MT-based, the rely on relayed exchange processes from membrane lipids to surrounding bulk water and, therefore, it can be difficult to determine the molecular origin of disease-based tissue contrast changes. NOE can help to separate the molecular origin of the MT effect from the lipid pool and bulk water, giving more molecular specificity.

Therefore, the purpose of this study is to utilize NOE imaging at 7T in conjunction with a five-pool Lorentzian line fitting model to investigate the lesion and normal-appearing tissue contrast differences between multiple sclerosis and healthy subjects in comparison to established anatomical structural imaging to demonstrate a metabolic profile between the two groups.

## Materials and methods

### Subject participants

Images were acquired on 15 multiple sclerosis subjects (10 females, five males; 11 relapsing–remitting multiple sclerosis, three radiologically isolated syndrome, one secondary progressive multiple sclerosis) and 10 healthy control subjects (five females and five males); recruited between February and December of 2023. Radiologically isolated syndrome subjects were diagnosed and included in the multiple sclerosis cohort based on 2017 MAGNIMS criteria.^23^ The average age of the multiple sclerosis subjects was 43.7 years (21–70 years), while the average age of the healthy controls was 40.9 years (23–71 years). All multiple sclerosis participants were recruited from the University of Pennsylvania Department of Neurology, Multiple Sclerosis and Related Disorders Outpatient Clinic. All subjects met the 2017 McDonald’s criteria for dissemination in space and time, except for one subject who met dissemination in space only. This subject did have central vein sign positive lesions and at least one paramagnetic rim lesion, which are both imaging biomarkers with high specificity to multiple sclerosis. Four subjects were not on a disease-modifying therapy at the time of scanning, while 11 were on a high-efficacy disease-modifying therapy, such as Ocrevus (seven subjects), Kesimpta (three subjects), or Mavenclad (one subject). Disease duration defined as the amount of time since the onset of clinical symptoms, or first observation of MRI finding concerning inflammatory demyelination, which was on average 6.5 years. All images were acquired after obtaining written informed consent under an approved University of Pennsylvania Institutional Review Board protocol. Subject demographic data described here can also be seen summarized in Table 1.

**Table 1:** Summarized subject demographic data

	Multiple sclerosis	Healthy control
No. of subjects	15	10
Sex	10 Female and 5 Male	5 Female and 5 Male
Average age (years)	43.7 ± 14.7	40.9 ± 16.9
Age range (years)	21–70	23–71
Disease duration (years)	6.5 ± 6.6	-
Average lesion volume (cm^3^)	1.2 ± 1.4	-
Disease subtype	11 RRMS, 3 RIS, 1 SPMS	

A table showing demographic data of both the multiple sclerosis and healthy control subjects imaged. It should be noted that lesion volume was calculated only from the effective 20 mm imaging slab and not across the whole brain volume. Disease subtypes listed as relapsing–remitting multiple sclerosis (RRMS), radiologically isolated syndrome (RIS), secondary progressive multiple sclerosis (SPMS).

### MR imaging acquisition

All images were acquired on a 7T system (MAGNETOM Terra, Siemens Healthcare, Erlangen, Germany) using a single-channel transmit 32-channel receive phased array head coil (Nova Medical, Wilmington, MA, USA). Image data were acquired via a modified volumetric (3D) turbo FLASH sequence, with gradient echo readout, TR = 3.9 ms, TE = 1.79 ms, shot TR = 6 s with three shots per image.^24^ A B_1,RMS_ amplitude of 0.72 µT was used to achieve magnetization preparation, with a saturation duration of 3 s. Thirty 99-ms duration Hanning-shaped pulses with a 1-ms inter-pulse delay were applied at frequency offsets ranging from −5 to 5 ppm with a step size of 0.2 ppm relative to the resonance of water proton, along with a 100-ppm saturation offset acquisition. These offsets were used to generate a full *in vivo* Z-spectrum which was then used in the Lorentzian fitting model. The acquisition had an in-plane resolution of 1 mm × 1 mm with a matrix size of 240 × 180. In the case of multiple sclerosis subjects, the 3D slab (24 mm thick, 2 mm per slice resolution) was positioned in an oblique orientation in the region of the brain with the highest number of visible lesions, generally superior to the corpus callosum. The first and last slices were excluded from the analysis due to aliasing artefacts. Therefore, the effective slab for analysis in both controls and multiple sclerosis subjects was 20 mm. Control subjects had the same 3D slab placed immediately superior to the corpus callosum. FLAIR and MP2-RAGE^25^ images with 0.7-mm isotropic resolution were also acquired as reference anatomical comparison images. The total time in this protocol was approximately 1 h. Additionally, 18 cm × 18 cm calcium titanate dielectric pads (7TNS, Multiwave Imaging, Marseille, France) were placed on either side of the head during acquisition to aid in correcting for B_1_^+^ inhomogeneities in the CEST images.^26,27^

### Postprocessing and Lorentzian fitting

Prior to fitting, denoising on the CEST-based images was performed by using a 4-dimensional Bloch Matching (BM4D) filter.^28^ A five-pool Lorentzian model was fit pixel-by-pixel to each Z-spectrum to derive a five-pool model, which included: direct saturation (DS), semi-solid MT, amide (APT), amine, and relayed NOE (rNOE).^16^ This model, given in Eq. 1, utilized the Levenberg–Marquardt algorithm and was parallelized in MATLAB to decrease fitting computation time:


(1) $$\frac{M_{Z}(\Delta \omega )}{M_{Z}^{0}(\Delta \omega )}=Z(\Delta \omega )=Z_{\mathrm{Base}}-\underset{i}{\sum }\;L_{i}(\Delta \omega )$$

where each individual Lorentzian function (*L_i_*), as seen in Eq. 2, can be characterized by offset frequency (*Δω*), amplitude (*A_i_*), full width half maximum value (*Γ_i_*), and chemical displacement relative to water protons (*δ_i_*). It should also be noted that in this model the fitting baseline parameter (*Z*_Base_) was fixed to a constant amplitude of 1 to better correct for a constant signal reduction.


(2) $$L_{i}(\Delta \omega )=A_{i}\left(\frac{\Gamma_{i}^{2}/4}{\Gamma_{i}^{2}/4+{\left(\Delta \omega -\delta_{i}\right)}^{2}}\right)$$

As an additional comparison, nuclear Overhauser effect magnetization transfer ratio (NOE_MTR_) was also calculated by normalizing the acquired −3.5 ppm signal to the 100 ppm signal as seen below in Eq. 3, where *S_0_* is the signal intensity of the 100 ppm signal.


(3) $$\mathrm{NO}E_{\mathrm{MTR}}=\frac{S_{0}-S(-3.5\mathrm{ppm})}{S_{0}}\times 100$$

Resulting images used a water saturation shift referencing acquisition, acquired at offset frequencies ranging from −1 to 1 ppm in step sizes of 0.1 ppm, to correct for the B_0_ inhomogeneities.^29^ A linear interpolation B_1_^+^ postprocessing step was applied to the acquired images to further assist in recovering contrast in low B_1_ magnitude areas, using B_1_ field maps as generated in Volz *et al.*^30^ The B_0_ correction was performed via local interpolation to a second order polynomial to eliminate off-resonance effects.^16^ To assess tissue-based contrast difference between groups, grey matter (GM) and WM segmentations were generated via T_1_ maps.^31^ Multiple sclerosis lesion specific maps were masked via manual identification on a 0.7 mm isotropic T_1_ map generated via the MP2-RAGE acquisition if lesions were visualized on both the corresponding FLAIR and T_1_ map images.^25^ Manual segmentations were performed using ITK-SNAP^32^ and registered to the corresponding volumetric NOE slab. Tissue and lesion volumes were estimated by multiplying the corresponding mask voxel count by the voxel resolution.

### Statistical analysis

Mean and standard deviation values were calculated for different contrasts within the slab for whole brain, GM, WM, and lesion tissue. Two-sample *t*-tests were used to compare whole brain and GM contrast levels between healthy controls and multiple sclerosis subjects. To adjust for subject age and sex, multiple linear regression was performed with contrast as the outcome and patient group (i.e. control versus multiple sclerosis) as the primary predictor of interest. To assess whether contrasts are significantly different between control WM, NAWM, and WM lesions, multiple pair-wise comparison *t*-tests with Bonferroni-correction were used. A linear regression model was used to assess the relationship between clinical disease duration and lesion load (volume) for each imaging contrast. All statistical analyses were performed using R Statistical Software (v4.3.1), and a significance level of *α* = 0.05 was used for all statistical tests.

## Results

**Figure 1:**
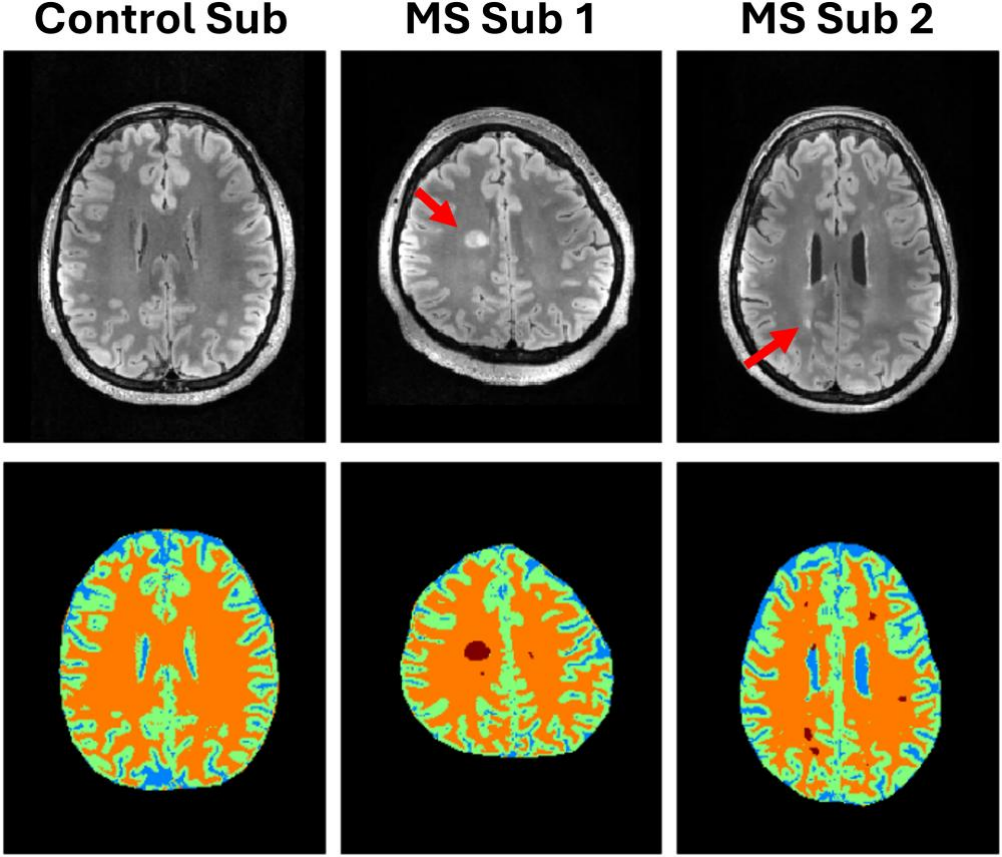
**Representative structural images and segmentations.** A representative set of FLAIR images (top row) for a healthy control subject and two multiple sclerosis subjects, in which prominent lesions can be seen in both subjects (red arrows). Example segmentations (bottom row) of grey matter, white matter, CSF, and identifiable lesions.

**Figure 2:**
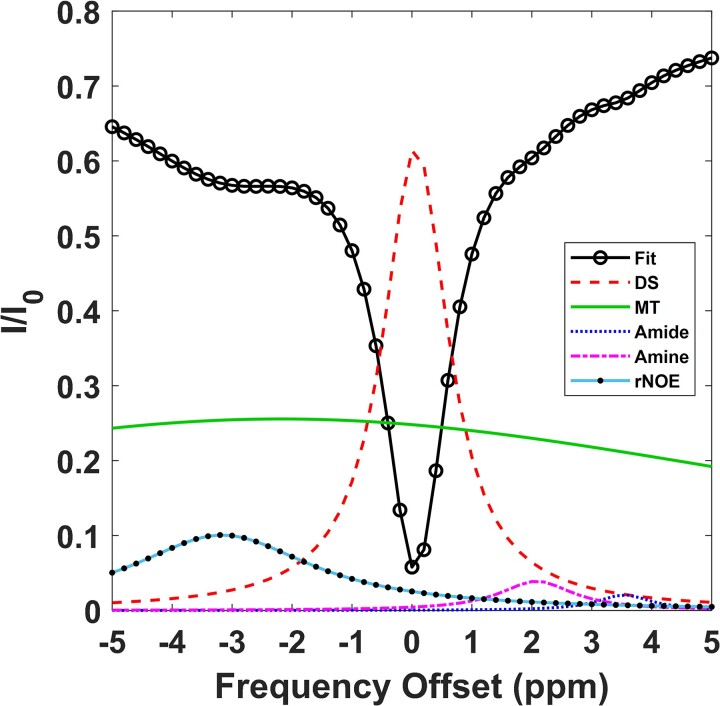
**Example whole brain Z-spectrum and multi-pool fitting.** A representative Z-spectrum fit (solid curve with open circles) from a healthy control subject with the corresponding pool fit peaks overlaid. The curves are shown in the following order: DS centred at 0 ppm (dashed curve), MT centred at −2 ppm (solid curve), amide centred at 3.5 ppm (dotted curve), amine centred at 2.2 ppm (semi-dashed curve), and rNOE centred at −3.5 ppm (solid-dotted curve).

The top row of Fig. 1 shows a qualitative comparison of FLAIR MR images between a representative healthy control subject and two multiple sclerosis subjects, with Subject 1 showing a prominent lesion. Additionally, an example of segmentation can be seen in the bottom row for these representative datasets in which GM is labelled as green, WM as yellow, CSF as blue, and the case of multiple sclerosis subjects, visible lesions in red. A representative Z-spectra can be seen in Fig. 2, with the corresponding pool fits averaged over the whole brain slab (GM and WM) of a healthy control subject. In particular, the rNOE peak can be seen centred at the −3.5 ppm frequency offset, while the other peaks DS, MT, amide, and amine can also be seen centred at 0, −2, 3.5, and 2.2 ppm, respectively.

**Figure 3:**
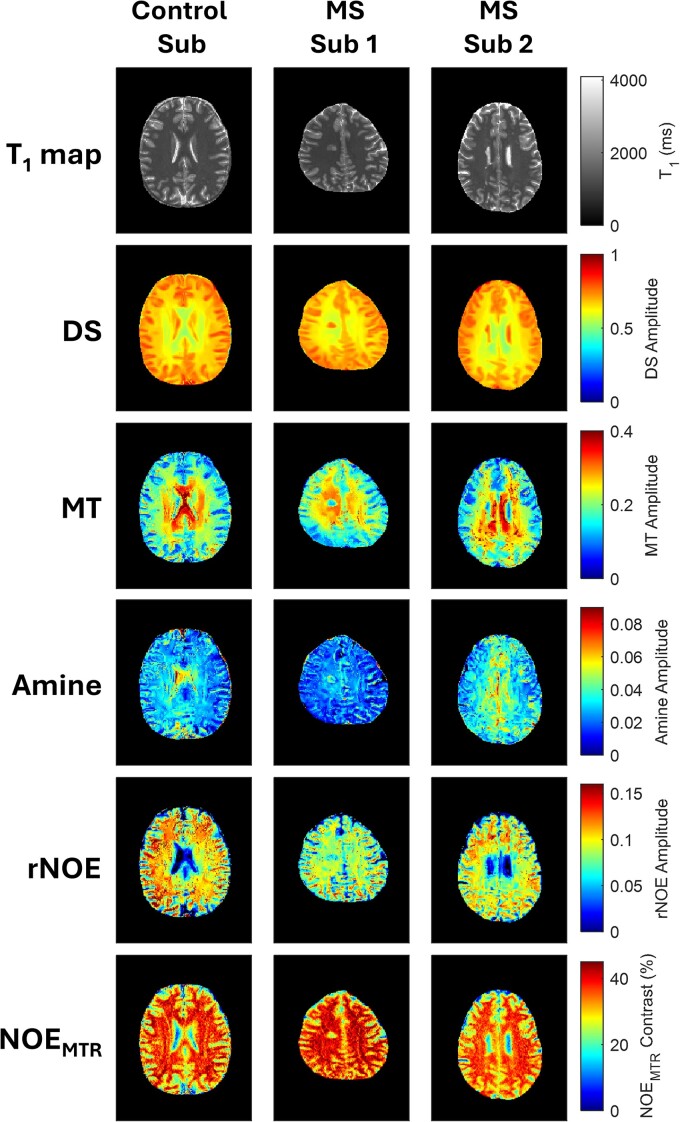
**Multi-pool fit image comparison.** A representative example image set from the same corresponding slice as in Fig. 1 showing a healthy control subject and two multiple sclerosis subjects. The first row shows conventional T_1_ maps with rows two through five showing DS, MT, amine, and rNOE pools generated via Lorentzian fitting. NOE_MTR_ contrast, shown in the last row, was derived using Eq. 3.

Figure 3 shows a representative acquisition and pool fitting example which includes T_1_ maps, DS, MT, amine, rNOE, and NOE_MTR_-weighted contrast images acquired on the same healthy and multiple sclerosis subjects seen previously. Changes in lesion contrast across many of the images and pool fits can be clearly observed compared to the normal-appearing tissue and healthy control tissue. More diffuse tissue contrast changes can be seen in the amine and rNOE pools as a pronounced contrast reduction in the central WM and cortical GM regions compared to the healthy control subject.

**Figure 4:**
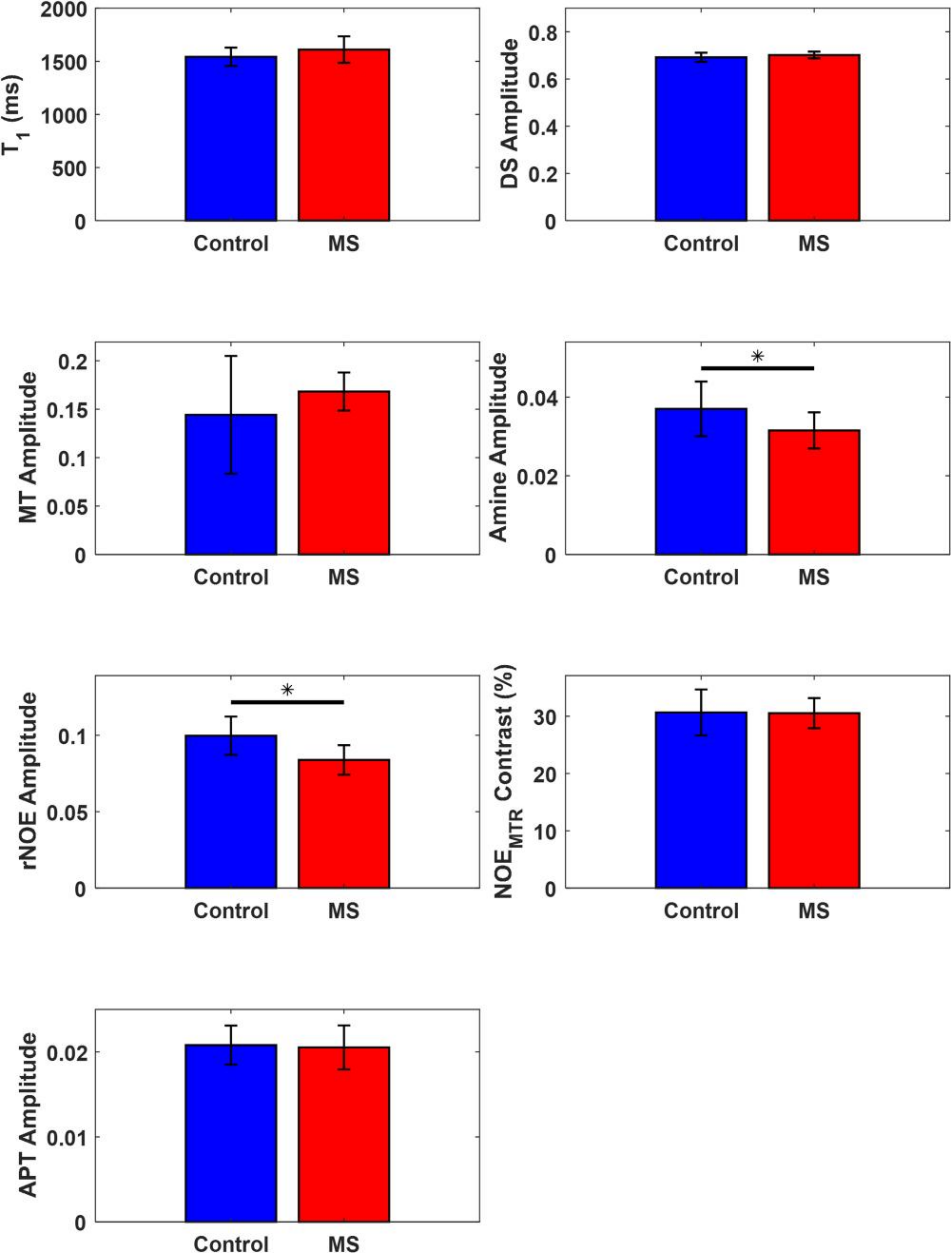
**Quantitative whole brain slab comparison.** Averaged whole slab imaging volume fit amplitudes (DS, MT, amine, rNOE, and APT) and contrast values (T_1_ and NOE_MTR_) between all multiple sclerosis (*n* = 15) and healthy control (*n* = 10) subjects. Two-sample t-tests were performed between healthy controls and multiple sclerosis subjects and when adjusted for age and sex dependencies, the amine and rNOE fits showed a statistically significant (amine: t-value = 2.19, *P* = 0.046; rNOE; t-value = 3.37, *P* = 0.004; *P* < 0.05 indicated by *) difference. The error bars within each plot are the standard deviation of the measured values.

Figure 4 shows a comparison of the average and standard deviation across all 15 multiple sclerosis subjects and 10 healthy control subjects for the whole brain imaging slab for the individual pool fit amplitudes (DS, MT, amine, rNOE, and APT), the T_1_ map, and NOE_MTR_-weighted contrast image. After adjusting for subject age and sex dependencies, statistically significant changes were observed in the amine pool with an average decrease from 0.037 ± 0.006 to 0.031 ± 0.004 (14.8% decrease) and in the rNOE pool with an average decrease from 0.099 ± 0.012 to 0.083 ± 0.009 (16.1% decrease). All other whole brain slab comparisons showed no statistically significant differences.

**Figure 5:**
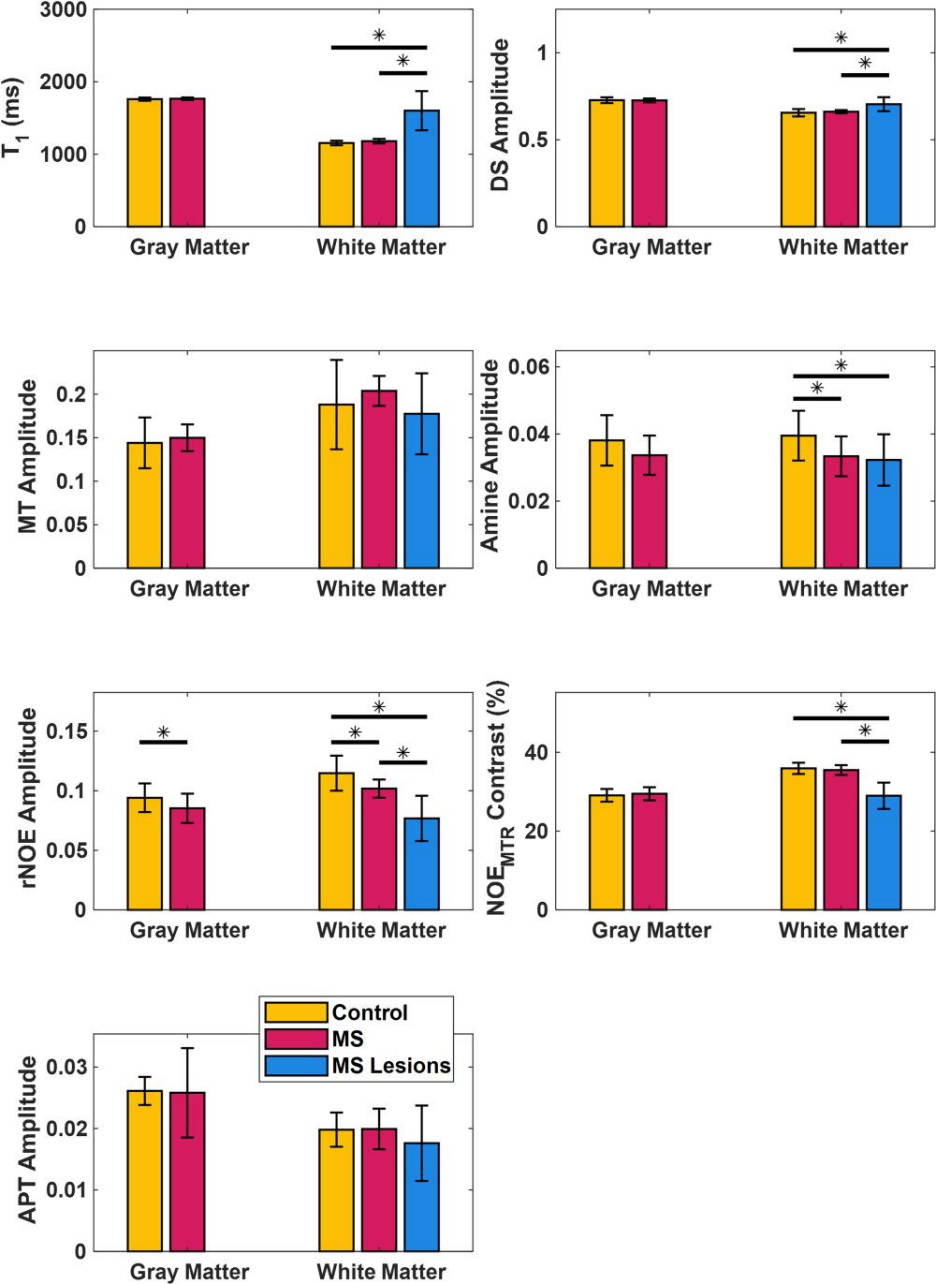
**Quantitative segmented tissue comparison.** Average and standard deviation fit amplitudes (DS, MT, amine, rNOE, and APT) and contrast values (T_1_ and NOE_MTR_) quantified across grey matter, white matter, and white matter lesion tissue types between multiple sclerosis (*n* = 15) and healthy control (*n* = 10) subjects. Bonferroni corrected multiple linear comparisons were performed and when adjusted for age and sex dependencies, statistically significant (*P* < 0.05 indicated by *) diffuse tissue contrast differences can be seen in the amine (NAWM: t-value = 2.29, *P* = 0.031) and rNOE (NAGM: t-value = 2.44, *P* = 0.028; NAWM: t-value = 2.88, *P* = 0.008) quantities, while lesion differences were present in T_1_ (Lesion/Control: t-value = −5.16, *P* < 0.001; Lesion/NAWM: t-value = −6.00, *P* < 0.001), DS (Lesion/Control: t-value = −3.50, *P* = 0.002; Lesion/NAWM: t-value = −4.02, *P* < 0.001), amine (Lesion/Control: t-value = 2.34, *P* = 0.028), rNOE (Lesion/Control: t-value = 5.31, *P* < 0.001; Lesion/NAWM: t-value = 4.71, *P* < 0.001), and NOE­_MTR_ (Lesion/Control: t-value = 6.19, *P* < 0.001; Lesion/NAWM: t-value = 7.08, *P* < 0.001). It should be noted that no significant differences were observed in the MT or APT pools.

Results seen in Fig. 5 show a segmented data comparisons between GM, WM, and lesion tissue types. There is a statistically significant difference in the rNOE amplitude between healthy control and multiple sclerosis GM after adjusting for age and sex, with an average decrease from 0.094 ± 0.012 to 0.085 ± 0.012 (10.6% decrease). After adjusting for age and sex, several statistically significance results were observed between the healthy WM and lesion tissue including an increase from 1154.6 ± 31.7 ms to 1600.7 ± 269.7 ms (38.6% increase) in the T_1_ value, a decrease from 0.039 ± 0.007 to 0.032 ± 0.007 (17.9% decrease) in the amine pool, an increase from 0.655 ± 0.021 to 0.703 ± 0.040 (7.3% increase) in the DS pool, a decrease from 0.114 ± 0.014 to 0.076 ± 0.019 (32.6% decrease) in the rNOE pool, and a decrease from 35.93 ± 1.43% to 28.97 ± 3.33% (19.3% decrease) in the NOE_MTR_-weighted contrast. When comparing NAWM and lesion tissue, statistically significant differences were observed including an increase from 1197.3 ± 31.8 ms to 1600.7 ± 269.7 ms (35.7% increase) in the T_1_ value, an increase from 0.660 ± 0.009 to 0.703 ± 0.040 (6.5% increase) in the DS pool, a decrease from 0.101 ± 0.007 to 0.076 ± 0.019 (23.9% decrease) in the rNOE pool, and a decrease from 35.49 ± 1.25% to 28.97 ± 3.33 (18.3% decrease) in the NOE_MTR_-weighted contrast. Comparisons between healthy WM and NAWM also showed statistically significant difference including a decrease from 0.114 ± 0.014 to 0.101 ± 0.007 (11.4% decrease) in the rNOE pool and a decrease from 0.039 ± 0.007 to 0.033 ± 0.006 (15.3% decrease) in the amine pool. All other tissue segmentation comparisons showed no statistically significant results. Tables summarizing the averages, standard deviations, percent changes, and corresponding statistical significance for whole brain, GM, and WM are shown in Supplementary Tables 1–3.

**Figure 6:**
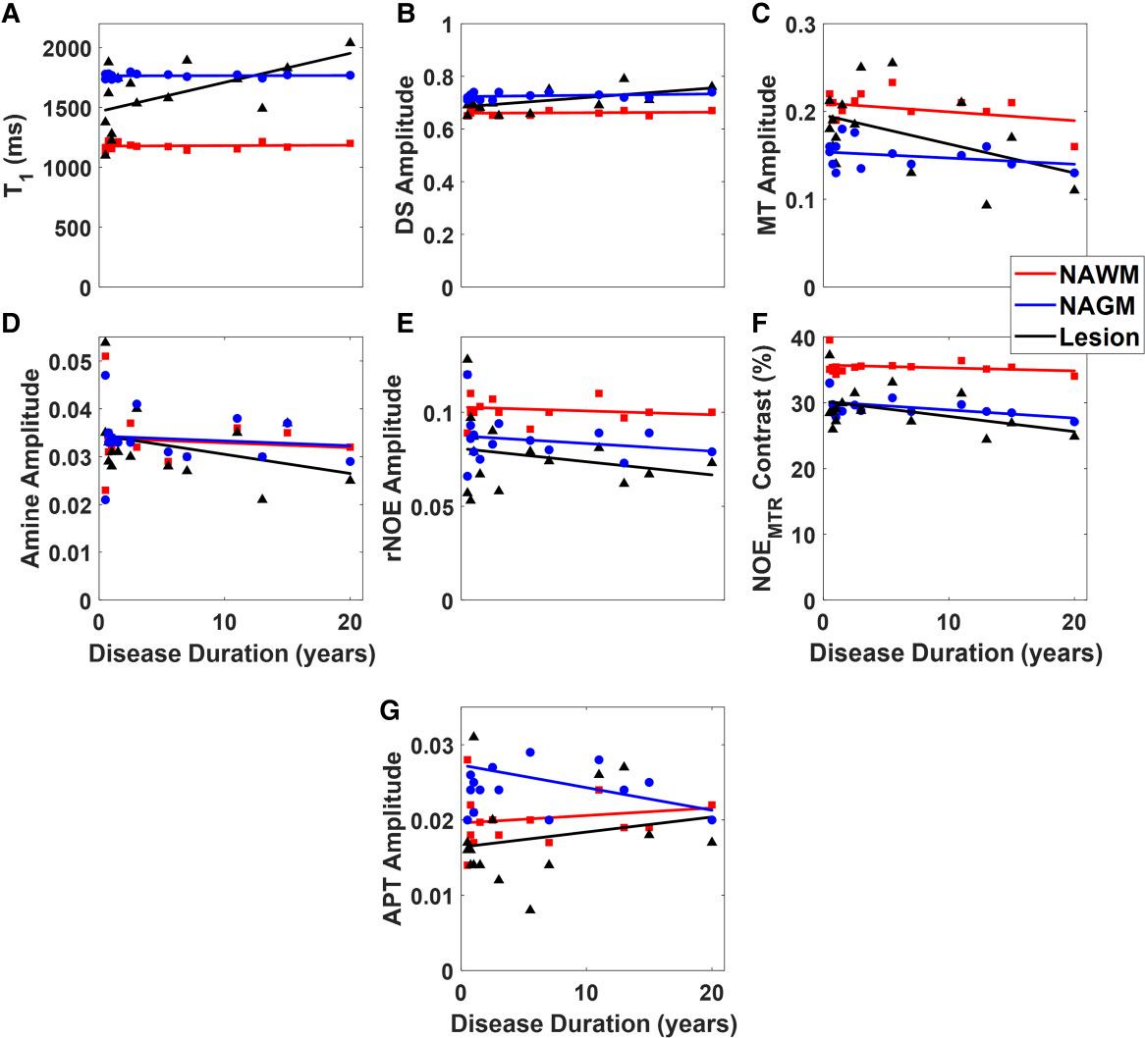
**Correlation between contrast and disease duration.** A series of plots showing the correlation between multiple sclerosis clinical disease duration and (**A**) T_1_ values (WM lesions: *β_1_* = 24.34, t-value = 2.49, *P* = 0.026), (**B**) DS amplitude (WM lesions: *β_1_* = 0.003, t-value = 2.46, *P* = 0.027), (**C**) MT amplitude (WM lesions: *β_1_* = −0.003, t-value = −1.82, *P* = 0.09), (**D**) amine amplitude, (**E**) rNOE amplitude, (**F**) NOE_MTR_-weighted contrast (NAGM: *β_1_* = −0.12, t-value = −1.92, *P* = 0.07; WM lesion: *β_1_* = −0.23, t-value = −1.74, *P* = 0.10), and (**G)** APT amplitude for NAWM, NAGM, and white matter lesions.

Figure 6 shows the relationships between the various image contrasts and clinical disease duration (years) exclusively in the multiple sclerosis subjects for NAGM, NAWM, and WM lesion segmentations. In Fig. 6A, a statistically significant positive correlation (*R^2^* = 0.32; *P* = 0.026) was observed between the T_1_ values and disease duration for the WM lesions. NAGM and NAWM T_1_ values both show no significant correlation to disease duration. Figure 6B shows a slight statistically significant positive correlation (*R^2^* = 0.32; *P* = 0.027) between the DS amplitude and disease duration in the WM lesions. NAGM and NAWM DS values both showed no significant correlation to disease duration. Figure 6C shows a negative trend (*R^2^* = 0.20; *P* = 0.09) between the MT amplitude and disease duration in the WM lesions, with the NAGM and NAWM showing no significant correlation. In Fig. 6D and E, both amine and rNOE amplitudes showed no significant correlations between any tissue type and disease duration. Figure 6F shows a negative trend towards statistical significance between the NOE_MTR_-weighted contrast and disease duration in both NAGM (*R^2^* = 0.22, *P* = 0.07) and WM lesions (*R^2^* = 0.19, *P* = 0.10) while NAWM showed no significant correlation. In Fig. 6F, no significant correlations were observed between APT amplitude and disease duration.

**Figure 7:**
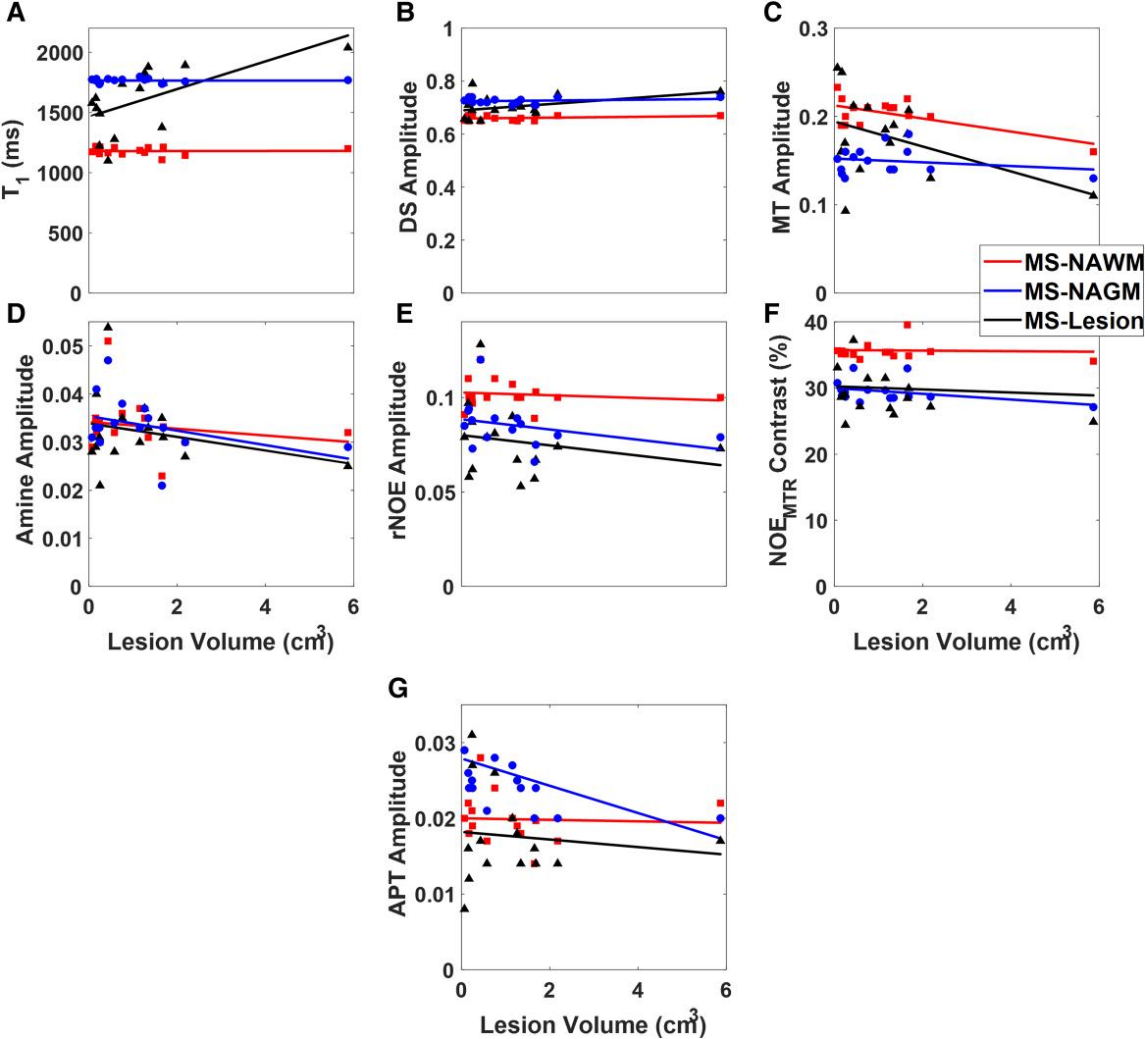
**Correlation between contrast and lesion volume.** A series of plots showing the correlation between multiple sclerosis lesion volume and (**A**) T_1_ values (WM lesion: *β_1_* = 0.003, t-value = 2.85, *P* = 0.01), (**B**) DS amplitude (WM lesion: *β_1_* = 15.66, t-value = 1.73, *P* = 0.10), (**C**) MT amplitude (NAMW: *β_1_* = −53.00, t-value = −2.88, *P* = 0.01; WM lesion: *β_1_* = −13.81, t-value = −1.77, *P* = 0.09), (**D**) amine amplitude, (**E**) rNOE amplitude, (**F**) NOE_MTR_-weighted contrast, and (**G**) APT amplitude for NAWM, NAGM, and white matter lesions.

Figure 7 shows the relationship between the various image contrasts and lesion volume exclusively in the multiple sclerosis subjects for NAGM, NAWM, and WM lesions. In Fig. 7A, a statistically significant positive correlation (*R^2^* = 0.38; *P* = 0.01) was observed between the T_1_ value and lesion volume for the WM lesion segmentation. NAGM and NAWM T_1_ values both show no significant correlation to lesion volume. Figure 7B shows a positive trend towards statistical significance (*R^2^* = 0.18; *P* = 0.10) between the DS amplitude and lesion volume in the WM lesion segmentation. NAGM and NAWM DS values showed no significant correlation to lesion volume, Fig. 7C, shows a statically significant negative correlation (*R^2^* = 0.39; *P* = 0.01) between the MT amplitude and lesion volume in the NAWM. Additionally, a negative trend towards statical significance (*R^2^* = 0.19; *P* = 0.09) was also seen between the MT amplitude and lesion volume in the WM lesion segmentations. In Fig. 7D–F, or 7 g no statistically significant correlation was seen for amine, rNOE, NOE_MTR_, or APT, respectively. An example APT pool map between a representative multiple sclerosis and healthy control subject can be seen in Supplementary Fig. 1. An additional correlation between MT and rNOE was performed, showing no strong relationship (R^2^ < 0.1) between the two contrasts in the multiple sclerosis subjects. This can be seen in as Supplementary Fig. 2.

## Discussion

NOE imaging was performed at 7T in conjunction with multi-pool Lorentzian line fitting to investigate tissue contrast changes between a cohort of multiple sclerosis and healthy control subjects. By using a pixel-by-pixel multi-pool Lorentzian line fitting, contributions from larger competing effects such as DS and MT, were separated, allowing us to produce specific spatial metabolite pool maps across a volumetric slab. Multiple sclerosis consists of a complex biology including focal inflammatory demyelination and chronic, widespread neuroinflammation as well as neuronal and glial degeneration. After accounting for age and sex dependencies, several statistically significant contrast changes in the WM lesion tissue were observed in comparison to NAWM and healthy control tissue in many of the pool fits including increases in T_1_ value and DS amplitude as well as decreases in amine amplitude, rNOE amplitude, NOE_MTR_-weighted contrast. Statistically significant diffuse changes were also seen in NAWM and NAGM as decreases in the amine and rNOE pools in comparison to healthy controls.

Several statistically significant positive correlations were also observed for disease duration which include the T_1_ value as well as the DS amplitude in the WM lesion tissue. Other comparisons also showed a similar trend towards significance such as MT amplitude and NOE_MTR_-weighted contrast in the WM lesion tissue and NOE_MTR_ in the NAGM. Similar trends were observed when contrast was correlated to the total lesion volume within the acquired slab which included the T_1_ value in the WM lesion tissue. Other comparisons also showed a statistically significant decrease in MT associated with lesion volume for the NAWM. WM lesion tissue showed the most numerous changes across contrasts, reflecting visible focal demyelination and diffuse neuronal injury. The increases seen in the DS pool correspond to an increase in tissue water content, while decreases in amine, rNOE, and NOE_MTR_-weighted contrast correspond to decreases in protein content, mobile lipids, and myelin-associated lipids, respectively.^33,34^ It should be noted that an analysis focusing on cortical GM lesions was not included due to the complex identification and segmentation process associated with these subtle lesions.

In addition to focal lesion contrast changes, we also observed significant changes in the NAGM and NAWM in multiple sclerosis subjects compared to WM and GM in healthy controls. These changes included a substantial decrease in the rNOE pool for both NAGM (10.6% decrease) and NAWM (11.4% decrease) as well as a decrease in the amine pool for NAWM (15.3% decrease). These diffuse tissue changes were only quantifiable in these two pools while changes in the conventional T_1_ maps showed minimal differences. Decreases in these pools reflect a reduction in signal from amine functional groups on proteins^35^ as well as decreases in mobile lipids,^33^ such as those found on neuronal cell body membranes, particularly in WM axon tracts. This indicates that not only is there substantial demyelination in the visible lesion areas but also that more subtle demyelination may be occurring in the WM and GM tissues. The results here may hold a measure of more diffuse tissue injury relating towards the chronic neurodegenerative disease process, which cannot be observed on conventional MRI. Neuronal injury caused by chronic inflammation in the NAWM and more extensive atrophy in the NAGM may also be linked to global non-lesional changes.^11,36^ These changes are qualitatively reflected in Fig. 3, where a representative example comparison between two multiple sclerosis subjects and a control subject are shown. Patient Subject 1 showed substantial visible decrease in the rNOE and amine pool contrasts for the central WM regions. This is also apparent in the GM regions which also show a notable contrast reduction. This visible change is not apparent from the T_1_ maps, in which the only discernable visual difference is in the lesions themselves rather than the diffuse tissue. One potential explanation behind the lack of quantifiable changes in the T_1_ maps between multiple sclerosis and healthy control subjects may be because most of the multiple sclerosis cohort in this work were diagnosed with relapsing–remitting multiple sclerosis, which can present as milder disease alterations (less pronounced tissue degeneration and reparation processes) reflected in the T_1_ values compared to subjects with more progressive forms.^37-39^ Additionally, conventional anatomic contrasts produced by the FLAIR sequence, seen in Fig. 1, also only show lesion contrast differences rather than global white and GM involvement.

Previous experiments, such as those performed by Huang *et al.,*^19^ utilized rNOEw contrast at 3T to investigate differences in lesion and diffuse tissue for multiple sclerosis and neuromyelitis optica spectrum disorder subjects compared to healthy controls. The authors observed decreases in rNOEw contrast for both the lesion and diffuse tissue in both populations, which agrees with the results presented here. The authors suggest that this decrease in the rNOEw signal is potentially associated with a decrease in myelin and the various associated protein concentrations present due to the extensive regions of demyelination. However, several confounding factors exist in their work, such as rNOEw contrast being the sole imaging biomarker investigated, which prevented the comparison of other relevant metabolite pools such as amine. Additionally, data acquisition at 3T field strength would produce inadequate chemical shift dispersion to effectively resolve the desired spectral peaks. Furthermore, B_1_ maps were not acquired in this previous work, therefore preventing the correction of any potential field inhomogeneities.

One of the main limitations in the current study is the lack of delineation between chronic and active lesions in the form of a separate analysis, due to gadolinium contrast acquisitions not being acquired. In the future, including an additional segmentation analysis between chronic and active lesions could provide more insight into heterogeneous pathophysiologic processes. Another limitation present was the relatively small sample size that precluded an assessment of sex differences. Larger sample sizes in future work would allow for this analysis. Also, the majority of the subjects imaged here had a diagnosis of relapsing–remitting multiple sclerosis, and therefore contrast differences between disease subtypes could not be properly assessed. A small subset of the subjects here had radiologically isolated syndrome which carries a higher risk of misdiagnosis, as not all radiologically isolated syndrome diagnoses will convert into multiple sclerosis. With few studies tracking longitudinal lesion or diffuse tissue changes in multiple sclerosis subjects using advanced MRI-based techniques,^40^ the parametric imaging described here could allow for a better understanding of the disease progression in a metabolite level. Additionally, tracking the effects of long-term disease modifying therapies may also prove useful as well as provide correlations between established CSF markers (such as NfL, GFAP, and IgG) and the contrasts investigated here. Finally, using the information derived from the incorporation of a similar CEST-based technique, glutamate-weighted CEST,^41^ may allow for more complementary information to aid in the early diagnosis of this condition. Future studies will focus on longitudinal tracking and investigation of other demyelinating diseases, such as genetic and acquired leukoencephalopathies.

## Supplementary Material

fcaf043_Supplementary_Data

## Data Availability

Anonymized subject data outlined in this study are available from the corresponding author, upon reasonable request.
