# Supplementary material for: Diffuse nuclear Overhauser effect MRI contrast changes detected in multiple sclerosis subjects at 7T
Source: Brain Commun. 2025 Feb 20;7(1):fcaf043. doi: 10.1093/braincomms/fcaf043 (PMC11840165; doi:10.1093/braincomms/fcaf043)
Supplement: fcaf043_Supplementary_Data [file fcaf043_supplementary_data.docx]

**Supplemental Material**

**Supplementary Table 1. Quantified Whole Brain Contrast Changes**

| **Whole Brain** | | | |
| --- | --- | --- | --- |
|  | **Control** | **Multiple Sclerosis** | **% Change** |
| **T_1_ (ms)** | 1542.1 (86.68) | 1610.3 (124.62) | 4.4 |
| **DS** | 0.692 (0.019) | 0.701 (0.014) | 1.3 |
| **MT** | 0.144 (0.060) | 0.168 (0.019) | 16.6 |
| **Amine** | 0.037 (0.006) | 0.031 (0.004) | -14.8* |
| **rNOE** | 0.099 (0.012) | 0.083 (0.009) | -16.1* |
| **NOE_MTR_ (%)** | 30.65 (4.01) | 30.52 (2.63) | -0.4 |

A table showing the average and standard deviation values for each contrast and fit amplitude across the whole brain slab. Percent change is also shown with (*) indicating comparisons that were statistically significant (*P* < 0.05).

**Supplementary Table 2. Quantified Gray Matter Contrast Changes**

| **Gray Matter** | | | |
| --- | --- | --- | --- |
|  | **Control** | **NAGM** | **% Change** |
| **T_1_ (ms)** | 1759.0 (21.92) | 1764.9 (18.24) | 0.3 |
| **DS** | 0.727 (0.016) | 0.725 (0.011) | 0.2 |
| **MT** | 0.144 (0.029) | 0.149 (0.015) | 3.4 |
| **Amine** | 0.038 (0.007) | 0.033 (0.005) | -13.1 |
| **rNOE** | 0.094 (0.012) | 0.085 (0.012) | -10.6* |
| **NOE_MTR_ (%)** | 29.08 (1.61) | 29.47 (1.67) | 1.3 |

A table showing the average and standard deviation values for each contrast and fit amplitude across the segmented gray matter regions. Percent change is also shown with (*) indicating comparisons that were statistically significant (*P* < 0.05).

**Supplementary Table 3. Quantified White Matter Contrast Changes**

| **White Matter** | | | | | | |
| --- | --- | --- | --- | --- | --- | --- |
|  | **Control** | **NAWM** | **Lesion** | **% Change (Control/NAWM)** | **% Change (Control/Lesion)** | **% Change (NAWM/Lesion)** |
| **T_1_ (ms)** | 1154.6 (31.72) | 1179.30 (31.81) | 1600.7 (269.75) | 2.1 | 38.6* | 35.7* |
| **DS** | 0.655 (0.021) | 0.660 (0.009) | 0.703 (0.040) | 0.7 | 7.3* | 6.5* |
| **MT** | 0.188 (0.051) | 0.203 (0.017) | 0.177 (0.046) | 7.3 | -6.2 | -12.8 |
| **Amine** | 0.039 (0.007) | 0.033 (0.006) | 0.032 (0.007) | -15.3* | -17.9* | -3.0 |
| **rNOE** | 0.114 (0.014) | 0.101 (0.007) | 0.076 (0.019) | -11.4* | -32.6* | -23.9* |
| **NOE_MTR_ (%)** | 35.93 (1.43) | 35.49 (1.25) | 28.97 (3.33) | -1.2 | -19.3* | -18.3* |

A table showing the average and standard deviation values for each contrast and fit amplitude across the segmented white matter regions. Percent change is also shown with (*) indicating comparisons that were statistically significant (*P* < 0.05).


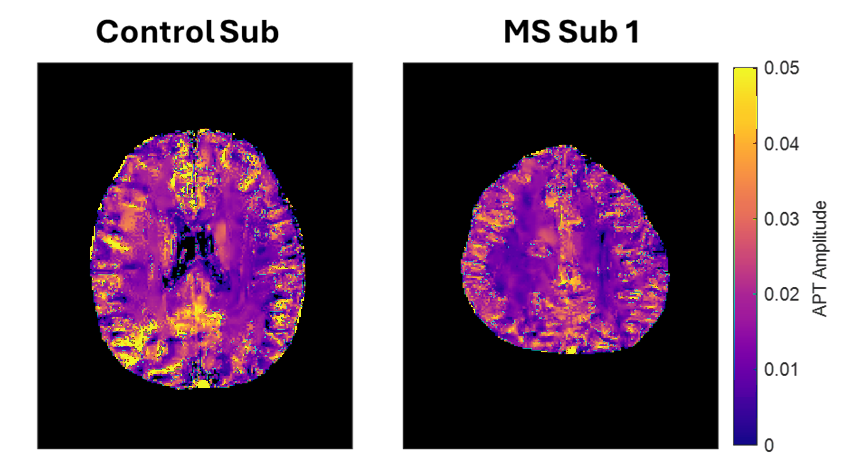


**Supplementary Figure 1.** Amide proton transfer (APT) amplitude contrast maps seen for a control and MS subject 1 showing minimal qualitative differences.

**Supplementary Figure 2.** Correlations between rNOE and MT amplitudes for both MS subject and healthy control data.
